# Supplementary material for: Are carbon emissions trading and green financial instruments synergistic? -Comprehensive quantitative research based on content analysis
Source: PLoS One. 2024 Mar 7;19(3):e0298601. doi: 10.1371/journal.pone.0298601 (PMC10919601; doi:10.1371/journal.pone.0298601)
Supplement: S1 Data — (ZIP) [file pone.0298601.s001.zip › Data/地级市Z_F工作报告.zip!/╣π╬≈/┴°╓▌╩╨/└┤╫╘╣1⁄2╓┌║┼í╛┬φ┐╦╩2╛▌═°í┐_2019.docx]

各位代表：

现在，我代表市人民政府向大会作政府工作报告，请予

审议，并请市政协委员和列席会议的同志提出意见。

一、2018 年工作回顾

2018 年，在自治区党委、政府和市委的正确领导下，我

们认真贯彻落实党的十九大和十九届二中、三中全会精神，

按照新发展理念和高质量发展要求，深入实施“实业兴市，开

放强柳”战略，全力推进“三大建设”，持续营造“三大生态”，

全市经济社会持续健康稳定发展。

——产业规划上升为自治区发展战略。自治区党委、政

府专门召开推进柳州市工业高质量发展、建设现代制造城现

场会，从广西战略发展的高度出台了实施方案，发出号令、

集结力量，全方位支持柳州工业高质量发展，全面开启柳州

建设现代制造城、打造万亿工业强市的新征程。

——经济发展平稳向好。初步预计，全市地区生产总值

突破 3000 亿元、达 3083.7 亿元，增长 7.0%；规模以上工业

增加值 1409.5 亿元，增长 3.6%；固定资产投资增长 15%；

财政收入 436.2 亿元，增长 8%，其中：一般公共预算收入

- 2 -

193.8 亿元，增长 7.8%；一般公共预算支出 424.38 亿元，增

长 13.3%。

——转型升级步伐加快。柳州实施老工业基地调整改造

成效显著，获国务院第五次大督查通报表扬。在自治区成立

60 周年庆祝大会上，中央政治局常委、全国政协主席、中央

代表团团长汪洋同志代表党中央作讲话时指出“柳州等老工

业基地脱胎换骨”，这是对柳州实施老工业基地调整改造的

充分肯定。三次产业结构为 6.5：52.9：40.6。预计第三产业

增加值增长 12%，对经济增长贡献率达到 66.4%。战略性新

兴产业产值占比提高到 13%，新增规模以上工业企业 93 家，

预计全市工业投资增长 10%，新兴动能加快成长。

——发展质量稳步提升。规模以上工业利税、利润分别

增长 14.6%和 36.3%，高于全国、全区平均水平。财政收入

质量提高，非税收入占一般公共预算收入比重 29.5%。

——生态优势持续巩固。市区空气质量优良率为 88.5%，

同比提高 4.1 个百分点；地表水水质优良率、市县集中式饮

用水水源地以及重要江河湖泊水功能水质达标率均为

100%。单位 GDP 能耗下降 2.5%。

——人民生活不断改善。城镇居民人均可支配收入

34784 元，增长 6.5%；农村居民人均可支配收入 13488 元，

增长 11%。顺利完成自治区下达脱贫攻坚年度任务，年内实

现 63 个贫困村、99897 人脱贫。共投入民生资金 351.04 亿

元，占一般公共预算支出的 82.7%。自治区、柳州市为民办

实事任务顺利完成。

- 3 -

一年来，主要做了以下工作：

经济结构调整优化升级。认真落实国家、自治区“六稳”

部署，构建“5+5”产业发展新格局，经济发展稳中有进、

稳中提质。传统产业加快改造提升，汽车工业研发能力增强，

上汽通用五菱、东风柳汽新车型投放市场，东风柳汽迁建商

用车基地、联合电子二期竣工投产，五菱柳机 2.0T 发动机、

菱特动力 V6 发动机生产线建成试产。钢铁工业质量效益再创

新高，柳钢集团实现利润 103 亿元，品种钢比重达 62%。机

械工业深入实施“二次创业”，完成产值 446.9 亿元、增长

19.7%，海外工程机械销量增长超过 40%。战略性新兴产业发

展迅速，广西汽车集团新能源整车基地启动建设，智能电网

产业园、智能轨道交通产业园、工业机器人产业园开工建设，

PC轨道梁预制场、装配式建筑 PC 生产基地和钢构生产基地

竣工投产。科技创新能力持续提升，国家小微企业创业创新

城市示范工作扎实开展，新增 1 家院士工作站、163 家国家级

高新技术企业；全国质量强市示范建设成效显著，上汽通用

五菱被认定为国家技术创新示范企业、获中国标准创新贡献

奖，柳工、东风柳汽被认定为国家级质量标杆，科技型中小

企业数量居全区第一。园区经济发展势头良好，完成园区规

模以上工业产值 2890 亿元、占全市总量 53%，柳东新区、北

部生态新区形成产业互补、联动发展新格局。现代服务业快

速发展，新增 200 家限额规模以上服务业企业。柳州国家公

路货运枢纽柳东物流中心、传化公路港等项目进展顺利，成

功申报成为国家流通领域供应链试点城市。创建了 2 家国家

- 4 -

级、8 家自治区级企业工业设计中心。全市电商交易额突破

900 亿元，柳州螺蛳粉互联网日均销量突破 100 万袋，预计全

年袋装螺蛳粉产值超过 40 亿元。全面落实房地产市场调控政

策，房地产业保持平稳健康发展。在融水县召开柳州市第一

届旅游产业发展大会，着力提升旅游产业发展质量，4A 级景

区由 23 家增至 31 家，全市旅游总消费 606.22 亿元，增长

34.75%。现代农业发展取得新成效，全市农林牧渔业总产值

344.4 亿元，增长 4.9%。粮食生产总体稳定，“双高”糖料蔗基

地建设扎实推进，水果、桑蚕、茶叶、水产畜牧养殖等产业

持续发展。获批创建柳南区国家现代农业产业园、融安金桔

中国特色农产品优势区，新增 6 个自治区级现代农业核心示

范区。主要农作物耕种收机械化作业水平达 62%。

精准脱贫攻坚全面推进。财政投入专项扶贫资金 19.99

亿元，增长 22.9%。易地扶贫搬迁安置住房建设任务全部完

成，贫困县“5+2”、贫困村“3+1”特色扶贫产业布局基本形

成，所有贫困村集体经济年收入实现 3 万元以上，农村贫困

发生率降至 6%。贫困村 20 户以上自然村屯道路基本实现硬

化全覆盖，解决 6 万人口饮水安全问题，完成 1.01 万户农村

危房改造。湛江—柳州“携手奔小康”扶贫协作行动扎实开展，

累计帮扶到位资金 1.64 亿元。

生态宜居城市建设成效明显。“一主三新”建设扎实推

进。城市色彩规划颁布实施。白沙大桥、官塘大桥、莲花大

道、曙光大道建成通车。柳州（鹿寨）至南宁高速公路改扩

建顺利完工。国家园林城市、国家森林城市通过复核，全市

- 5 -

森林覆盖率 65%，城市建成区绿化覆盖率 43.95%，花园城市

2.0 建设圆满收官，展现了“紫荆花城·醉美柳州”“春花

秋水·画卷柳州”城市新形象。全国老旧小区改造试点工作

顺利完成。扎实开展清理整治违法建设“飓风行动”，拆除存

量违法建筑面积 594.5 万平方米。污染防治攻坚成效明显，

加强莲花山片区生态保护，“河长制”全面落实，中央环保

督察 2017 年反馈意见问题整改全面完成。

改革开放呈现崭新局面。供给侧结构性改革深入推进，

全年减免各项税费 101.5 亿元。深化国资国企改革，市属国

有资产总额突破 6000 亿元、达 6020 亿元，增长 6.5%。全市

私营企业及个体工商户达 26.6 万户，增长 20.8%。防范和化

解金融风险取得积极成效，银行业存贷款余额分别增长 2.4%

和 19.4%。启动农村集体产权制度改革，基本完成农村土地

承包经营权确权登记颁证工作。全力优化营商环境，深化放

管服改革，扎实推进“354560”改革，“一事通办”+实现率达

96.95%。开放步伐不断加快，成功举办第八届中国—东盟（柳

州）汽车工业博览会等国际性会议。首个国际航班正式开通。

全市进出口总额 181 亿元，增长 5%；引进市外境内到位资

金 879.8 亿元，实际利用外资 8 亿美元、增长 17.8%。

民生与社会各项事业实现新发展。大力推进“大众创业、

万众创新”，发放就业创业补贴 1.48 亿元、创业担保贷款 4.3

亿元。城镇新增就业 6.11 万人，农村劳动力转移就业 7.94

万人，城镇登记失业率 2.92%。市财政安排 1.6 亿元实施人

才强市战略，新时代人才集聚新政全面实施。社会保险覆盖

- 6 -

面不断扩大，城乡居民养老保险、医疗保险参保率分别达到

90.3%、97.5%。“15 分钟社区养老服务圈”启动建设，城乡

低保标准再次提高。超额完成保障性住房建设任务，棚户区

改造新开工建设 12216 套、基本建成 7047 套，公共租赁住

房分配入住 32030 套。安排 1 亿元实施少数民族村寨防火提

升改造工程。投入 7.7 亿元改善 335 所农村学校办学条件，

市区新建、改建学校 4 所，新增学位 9700 个。高考一本上

线率位居全区第一。全国“现代学徒制”−试点工作顺利完成，

职业教育国际化实现新突破。圆满完成自治区成立 60 周年

大庆各项工作。成功举办 2018 世界铁人三项赛、国际水上

狂欢节暨首届国际水上休闲运动会、环广西公路自行车世界

巡回赛柳州段、柳州国际奇石节等活动赛事。医改便民惠民

效果明显，公立医院、基层医疗机构服务能力增强，妇幼健

康指标稳步提升，疾病预防、卫生应急取得新成效。双拥共

建深入开展，军政军民团结更加巩固。深入推进平安柳州建

设，扫黑除恶专项斗争成效显著，创建广西食品安全示范城

市通过自治区验收，安全生产形势持续平稳，社会大局保持

和谐稳定。

过去一年，我们主动接受市人大及其常委会的法律监

督、工作监督和市政协的民主监督，继续办理 2017 年人大

议案 3 件、组织实施 2018 年人大议案 1 件，办理人大代表

建议 131 件、政协提案 221 件，市人大代表议案建议和市政

协委员提案办结率、满意率均为 100%。

过去一年，我们重视政府自身建设，始终在思想上政治

- 7 -

上行动上同以习近平总书记为核心的党中央保持高度一致，

严格落实中央“八项规定”精神和廉洁自律各项规定，以求实、

务实的态度接受国务院大督查，做好自治区党委第一巡视组

巡视反馈意见整改，持续纠正“四风”，不断强化行政监察和

审计监督，狠抓正风肃纪，努力打造人民满意政府。

一年来，全市人事、编制、绩效、外事侨务、税务、物价、

统计、国家安全、档案、地方志、气象、水库移民、供销、调

处、老龄、妇女儿童、红十字、残疾人事业、防汛抗旱、防震

减灾、社会科学等工作都取得了新成绩，作出了新贡献。

在看到成绩的同时，我们清醒认识到，当前发展不平衡

不充分的问题还比较突出，经济社会发展还面临诸多的困难

和挑战。主要是：推动高质量发展的基础还不够牢固，构建

产业新体系任重道远；脱贫攻坚任务艰巨；公共服务供给仍

然不足，教育、就医、养老等方面还需要进一步提升；节能

减排、环境保护形势依然严峻；行政效能有待进一步提高等。

针对这些问题，我们将竭尽全力加以改进和解决。

各位代表！过去一年，成绩来之不易。这是党中央、国

务院和自治区党委、政府正确领导的结果，是在市委带领下

团结一致、锐意进取的结果，是市人大、市政协监督支持和

帮助的结果，是全市各族人民齐心协力、拼搏奋斗的结果。

在此，我代表市人民政府，向全市各族人民，向驻柳部队官

兵，向各民主党派、工商联、人民团体以及所有关心、支持

柳州发展的各界人士，表示衷心的感谢并致以崇高的敬意!

- 8 -

二、2019 年工作安排

2019 年是新中国成立 70 周年，是决胜全面建成小康社

会第一个百年奋斗目标的关键之年。按照中共柳州市第十二

届代表大会第四次会议的工作部署，今年政府工作的总体要

求是：以习近平新时代中国特色社会主义思想为指导，深入

学习贯彻党的十九大和十九届二中、三中全会精神，以及中

央和自治区经济工作会议精神，坚持稳中求进工作总基调，

坚持新发展理念，坚持推动高质量发展，以供给侧结构性改

革为主线，深入实施“实业兴市，开放强柳”战略，大力开展

“四个年”活动，持续打好产业转型升级、基础设施建设和

精准脱贫三大攻坚战，全力推进“三大建设”，持续营造“三

大生态”，加快建设现代制造城，奋力实现在全区率先全面

建成小康社会，不断夯实打造西江经济带龙头城市和万亿工

业强市基础，以优异成绩庆祝中华人民共和国成立 70 周年。

2019 年柳州经济社会发展的主要预期目标是：地区生产

总值增长 7%以上，规模以上工业增加值增长 5.5%，固定资

产投资增长 13%，财政收入增长 6.5%，社会消费品零售总额

增长 10%，城镇居民人均可支配收入增长 7%，农村居民人

均可支配收入增长 10.5%。城镇登记失业率和单位生产总值

能耗、主要污染物排放总量控制在自治区下达指标以内。

上述预期目标的设定，主要基于以下方面考虑：一是按

照党中央、国务院关于稳中求进工作总基调和自治区全力以

赴稳住今年经济增速水平的要求，坚持把全力以赴稳增长作

- 9 -

为首要任务，确保经济运行在合理区间。二是与自治区预期

目标相对应，同时兼顾与柳州市“十三五”规划目标相衔接，

保证全市经济增速略高于国家和自治区的增速。三是预期目

标更好地体现了高质量发展要求，符合柳州当前发展的阶段

性特征，使我们能够把更多精力集中到优化调整经济结构和

转换增长动力上来。

围绕今年的目标任务，重点抓好以下几个方面的工作：

（一）开展经济发展提质年活动，加快推进工业高质量

发展

加快传统产业转型升级。举全市之力争创制造业高质量

发展示范区。推动汽车工业持续健康发展，紧盯形势发展变

化，未雨绸缪、抢抓机遇，加快转型升级。推进上汽通用五

菱、东风柳汽新车型研发，建设上汽通用五菱“跨十工程”、

一汽柳特整体搬迁改造升级等项目，建成东风柳汽研发试验

中心。深入推进汽车零部件再升级改造，实施广西汽车集团

佛吉亚扩能项目和上汽变速器公司新型变速器、发动机等项

目，补齐补强产业链。钢铁产业加快柳钢本部基地开展汽车

用钢、高附加值品种钢研发，实施柳钢特种钢精线深加工等

项目。深入推进机械产业“二次创业”，加快柳工工程机械智

能化改造升级。实施柳州卷烟厂百万箱技术改造、金嗓子洛

维生产基地等项目建设，支持两面针、柳化等企业优化发展

结构，提升产品竞争力。

发展壮大战略性新兴产业。推进广西汽车集团新能源整

车基地建设，加快引进新能源汽车电池、电机、电控等企业，

- 10 -

推广应用新能源城市用车。加快智能轨道交通产业发展，引

进一批优质企业落户智能轨道交通产业园。全力推进智能电

网产业园建设，加快一批全领域关键设备及软件研发制造企

业落地。探索装配式建筑产业发展模式，推广装配式建筑应

用，力争装配式建筑 PC 构件达到 12 万立方米生产能力。以

柳工、上汽通用五菱机器人应用为带动，推进北部生态新区

机器人产业园开园运营。大力发展铝深加工产业，打造产业

集群。以联合电子、耐世特等企业为龙头，加快培育电子信

息产业，推动龙腾光电、信炜科技等项目落地建设。以柳药、

花红药业为龙头，发展壮大生物与制药产业。

不断增强科技创新支撑能力。推进国家知识产权试点城

市、全国质量强市示范城市和柳州自主创新示范区建设，开

展 20 个重大科技专项、培育 20 个创新标杆企业，加快科技

成果转化。持续开展“大众创业、万众创新”，加快建设上海

漕河泾柳东创新创业园，做好国家小微企业创业创新基地城

市示范验收工作。深入实施柳州城市转型升级质量发展导

则，启动柳州国家汽车质量监督检验中心二期建设，力争国

家级高新技术企业超过 450 家。大力实施人才强市战略，落

实柳州加快新时代人才集聚措施，大力引进高端人才团队，

实施“万千学子入柳计划”。

大力发展民营经济。扩大中小企业（民营经济）发展专

项扶持资金规模，出台柳州市降低民营企业用地成本、物流

成本和政策性融资担保风险分担、代偿补偿等措施，全方位

支持民营经济发展壮大。依法保护民营企业的资产和权益，

- 11 -

支持民营企业科技创新、管理创新，建设非公服务平台、小

微企业创业创新服务中心、“柳创汇”创业创新云平台，加快

民营企业转型升级。

增强工业园区产业承载能力。进一步完善园区基础设施

建设，整体提升园区功能配套，增强园区为企业服务的能力。

大力推进园区土地储备与开发，加大产业招商力度，对入园

项目实行全程跟踪服务。加快阳和工业新区申报成为国家级

经济技术开发区，推动鹿寨经济开发区升级为自治区级高新

区。年内完成各县区工业园区规划审定工作，不断提高全市

园区产业集聚发展质量。

（二）促进第三产业提质增效，提升现代服务业新优势

大力发展生产性服务业。发展工业设计，推进与洛可可

合作项目建设，促进工业设计与产品创新深度融合，推动中小

企业产品改造升级。发展工业大数据，启动大数据产业园一期

建设，引进一批信息化企业落户园区，支持上汽通用五菱开展

智能网联合作。发展工业科研，建设中国科学院创新创业学院

柳州科技创新示范基地、电子科技大学广西智能制造产业技术

研究院、广西科技大学洛客设计学院等产学研平台，力争年内

全社会研发投入强度达到 2%以上。发展工业物流，建设国家

流通领域供应链试点城市，实施宁铁物流园、传化公路港、顺

丰、万科、中通等一批物流项目，打造高铁航空物流基地，推

动生产性服务业向专业化和价值链高端延伸。

进一步扩大消费需求。拓展生活性服务业，年内新增 120

家限额规模以上服务业企业。推动传统商圈转型升级，提升

- 12 -

万达、华润等商业综合体集聚效应，加快红星美凯龙、友邻

汇等商贸项目建设。促进汽车消费，培育新能源汽车消费市

场，推动新能源汽车推广应用“柳州模式”在广西推行，全力

扩大新能源汽车在全区的销量。实施“电商柳州”行动计划，

推进国家电子商务进农村综合示范县项目，引导企业入驻阿

里巴巴柳州产业带，支持外贸企业开展跨境电子商务。实施

柳州螺蛳粉“六个一”工程，建设食品（螺蛳粉）产业园，

构建螺蛳粉食品质量安全可追溯体系。开展农贸市场环境脏

乱差问题整治，实施城区 30 个以上农贸市场升级改造。坚

持房子是用来住的不是用来炒的定位，稳地价、稳房价、稳

预期，保持房地产市场健康发展。

建设现代金融服务体系。大力发展产业金融，推动科技

金融和绿色金融协同发展，着力打造区域性产业金融服务中

心。加快发展普惠金融，建设中小微企业信用信息平台，完

善中小工业企业应急周转资金管理办法，扩大“惠企贷”“银

税贷”业务覆盖范围，发挥银行业债权人委员会会商机制作

用，帮扶暂时经营困难企业解危解困。深化农村金融改革，

拓宽农村金融普惠网络。鼓励和支持上市公司利用上市平台

进行兼并重组等资本运作，积极推动有条件的企业加快上市

步伐。完善政府性融资担保体系，健全担保基金风险分担、

分散及补偿机制，吸收社会资本做大基金业务。严厉打击和

处置非法集资，营造金融市场健康发展环境。

加快发展旅游产业。编制全域旅游产业规划，主动融入

桂滇黔及粤港澳大旅游圈，建设融安、融水、三江少数民族

- 13 -

百里风情旅游带。推动融水县和城中区创建国家级全域旅游

示范区、鹿寨县创建广西特色旅游名县、柳江区创建自治区

级全域旅游示范区。全力支持三江县承办柳州市第二届旅游

产业发展大会，建设优质旅游项目。加快百里柳江、程阳八

寨创建国家 5A 级旅游景区，建设融水环元宝山民族生态旅

游区。大力发展特色民宿产业，培育旅游精品线路，打造智

慧旅游。

（三）全面实施乡村振兴战略，努力构建现代农业产业

体系

促进城乡融合发展。实施乡村振兴产业发展、基础设施、

公共服务能力提升三年行动计划，加快建设融安至从江高速

公路一期工程（融安至安太段），年内完成落久水利枢纽工

程下闸蓄水、库区移民安置，力争开工建设洋溪水利枢纽、

梅林航运枢纽工程。推进“四好农村路”建设，实施 42 个乡

乡通二级（三级）公路项目，实现 20 户以上自然村屯通屯

道路硬化和建制村通客车全覆盖。积极争创国家级特色小

镇，统筹整合各类项目、专项资金支持特色小镇建设。

加快现代农业发展。进一步稳定粮食生产，大力发展水

果、桑蚕、茶叶、中药材等特色农业，年内完成建设 46.4 万

亩“双高”糖料蔗基地，启动建设 84 万亩糖料蔗保护区。做好

非洲猪瘟防控，推进畜禽生态综合养殖、水产健康养殖。继

续争创国家、自治区特色农产品优势区和自治区级现代农业

核心示范区。进一步提高农业机械化水平。推进“三品一标”

认证，强化农产品（食品）加工、流通及质量安全监管。发

- 14 -

展“互联网+现代农业”，实现农村电商服务点进村全覆盖。

开展乡村风貌提升工作。实施乡村风貌提升三年行动，

推进“美丽柳州·幸福乡村”建设，开展乡村规划“三落实”、

特色风貌“三提升”、乡村文明“三治理”和实施村庄基础设施

“七改造”、村庄公共服务“十完善”行动，将乡村建设成为生

活舒适的乐园、生态良好的田园、乡愁记忆的家园。进一步

完善农村基础设施，推动“厕所革命”标准化、规范化建设，

美化村庄田园、村落庭院。开展传统村落保护发展立法，保

护建设融水良寨国里屯、三江独峒林略村和林溪高秀村 3 个

国家级传统村落。

促进县域经济健康发展。柳江区全面承接市区产业辐

射，提升园区配套水平，大力发展先进装备制造、电子信息

等产业。柳城县加快融入北部生态新区同城化发展，推进不

锈钢紧固件、食品深加工等产业园建设。鹿寨县推进现代林

业科技产业园、江口工业园、石墨烯新材料产业基地等项目

建设。融安县推动金桔、香杉等特色产业向品牌化、标准化、

规模化发展，建设香杉生态工业园、农产品加工园。融水县

大力发展全域旅游，加快元宝山—贝江、老子山—老君洞等

景区建设，做大做强竹木加工、生物与制药、健康食品等产

业。三江县充分发挥区域交通优势，进一步挖掘民族风情旅

游资源，发展“两茶一木、种稻养鱼”生态产业，争创三江茶

中国特色农产品优势区。（四）开展项目建设提速年活动，发挥有效投资的关键

性作用

- 15 -

集中力量推进项目建设。坚持把抓项目、扩投资作为高

质量发展的重要支撑，集中力量实施好 553 个市级层面重大

项目，力争年度完成投资 686.5 亿元。实施好 589 个城建项

目，力争年度完成投资 349 亿元。狠抓工业投入，围绕 233

个现代制造城项目建设，按年度作分解，列出施工图、计划

表，明确责任，确保年内全市工业投资增长 7%以上。

大力开展精准招商。实施产业招商五年行动计划，引进

一批行业龙头企业和高成长性企业，年内引进项目 230 个，

其中工业项目比重达到 60%以上。完善招商引资激励考评机

制和督查督促机制，健全“一把手”招商制度，发挥十大产业

招商组作用，开展珠三角、长三角专题招商活动，提高招商

引资成效，全年引进市外境内到位资金增长 7%，全口径实

际利用外资增长 10%。

强化项目建设要素保障。积极争取国家与自治区级财政

资金支持，引导银行信贷资金、民营资金投向重大项目建设。

完善重点项目用地保障机制，持续开展征地拆迁攻坚行动，

盘活存量土地，提高土地利用率。加快推进项目前期工作，

继续实行领导干部联系重点项目制度，开通重大项目审批“绿

色通道”，提高审批效率。

（五）开展精准脱贫攻坚年活动，全面完成年度脱贫工

作任务

确保现行标准下的脱贫质量。深入实施脱贫攻坚三年行

动计划，确保 2019 年减贫 7 万人以上、90 个以上贫困村脱

贫出列、融安县脱贫摘帽。围绕贫困户脱贫“两不愁三保障”

- 16 -

等工作，坚决打好精准脱贫攻坚战，推动脱贫攻坚取得更大

实效。

全力攻克深度贫困地区脱贫任务。全面落实中央、自治

区支持深度贫困地区脱贫攻坚政策，强化教育医疗住房“三保

障”，实施控辍保学攻坚，推进基层医疗卫生机构标准化建设，

力争所有贫困人口住房安全保障全部达标。打好脱贫攻坚“五

场硬仗”，发展贫困县“5+2”、贫困村“3+1”特色产业，创建

“三微”平台，确保完成 5.68 万人易地扶贫搬迁，年内所有

已脱贫和计划脱贫村的村集体经济年收入达到 4 万元以上。

继续推进湛江—柳州“携手奔小康”扶贫协作行动，办好贫困

群众急需的民生实事。

构建稳定脱贫、防范返贫的有效机制。开展脱贫攻坚“五

大专项行动”，做好就业意愿、就业技能与就业岗位精准对

接。健全脱贫综合保障体系，将符合条件的建档立卡贫困残

疾人纳入农村低保、城乡医疗救助、救助供养等保障范围。

加大金融扶贫工作，确保贫困县信贷增速高于全市平均水

平。深化“村企共建”“百企扶百村”工作，落实脱贫后续帮扶，

巩固脱贫成果。

（六）坚决打好污染防治攻坚战，共建天蓝地绿水净美

好家园

持续推进大气污染防治。以季节性大气污染防治攻坚为

重点，全面落实大气污染防治行动计划，开展扬尘治理、露

天禁烧、烟花爆竹禁放等工作。狠抓节能减排，强化工业企

业污染治理，提高清洁能源在全市能源消耗中的比重。

- 17 -

扎实推进水污染防治。全面推行“河长制”，开展“清

河行动”，实施小流域水污染防治综合治理，加强县级饮

用水水源地保护。实施城镇污水处理提质增效三年行动，建

设镇级污水处理设施及配套工程。构建竹鹅溪整治“长制久

清”工作机制，实现市区建成区生活污水全面收集处理。

精准推进土壤污染防治。大力推进自治区级土壤污染综

合防治先行区建设，认真开展土壤污染治理与修复工作。加

快推进静脉产业园、建筑垃圾循环利用项目建设，加强对涉

重金属企业的监管和执法。开展“清洁田园”专项活动，建立

清洁田园示范片 20 个，清洁技术推广面积 400 万亩。

构建生态文明建设新体系。大力发展生态环保产业，推

进国家循环经济示范城市、柳州国家第三批低碳试点城市建

设。全面加强柳江河流域生态环境保护，推进柳江河流域生

态环境保护立法工作。强化工业、交通、建筑等多领域协同

共治，积极创建国家级生态文明建设示范市。扎实做好莲花

山片区保护，启动保护区边界标识设置，将保护区范围扩展

至古亭山片区。

（七）提升城市综合承载能力，建设现代化的美丽宜居

柳州

进一步优化城市发展布局。继续做好第三次全国土地调

查工作，启动市县国土空间总体规划编制工作，完善片区控

制性详细规划，优化重点路段、主要景观地带城市设计。加

快“一主三新”城市建设，柳东新区进一步完善城市基础设

施，加大国家产城融合示范区建设力度；推进北部生态新区

- 18 -

“三纵三横”骨干路网等基础设施提速建设；大力实施柳江区

十大城市建设工程，提升柳江区规划管理水平。

完善城市综合功能。加快城市公共交通配套一、二期工

程建设，争取城市轨道交通示范线车辆上线。继续推进城区

体育公园项目建设，进一步完善“15 分钟步行生活圈”。实

施城市道路交通畅通工程，全线贯通城市快速环道，缓解城

市交通拥堵。深入实施城市“绿化、美化、亮化”工程，巩固

花园城市 2.0 建设成果。做好城市棚户区改造，开展老旧小

区整治、市区低洼积水片区排涝治理和海绵城市、城市“双

修”建设等工作。实施新型城镇化建设三年行动计划，有序

推进农业转移人口市民化，争取全市常住人口城镇化率达到

66.4%。

构建城市管理长效机制。加快智慧城市建设，实施数字

柳州建设三年行动计划，建成电子政务云二期、政务信息系

统整合共享平台，扩大大数据应用。着重从源头解决噪音扰

民问题，强化联合执法，严格考核问责。制定垃圾分类指南，

鼓励城市居民实行生活垃圾分类。深入开展清理整治违法建

设“飓风行动”，加大对闲置土地、批而未供用地处置。

（八）扎实开展营商环境突破年活动，着力打造成为营

商环境最优的现代化工业城市

深入开展优化营商环境大行动。实施进一步减轻企业税

费负担 24 条措施和工业用地弹性出让等政策，制度化、常

态化召开政策说明会，确保“1+13”优化营商环境政策落到

实处。引导金融机构加大对企业的信贷支持，降低企业融资

- 19 -

成本，缓解企业融资难、融资贵问题。完善社会征信体系，

建立联动机制，开展信用联合奖惩，建设诚信柳州。扎实开

展第四次全国经济普查工作。

打造优质高效的政务环境。深化“放管服”改革，全面

推行“一事通办”政务服务，深入推进“354560”“证照分离”=

等专项改革，完善“一枚印章管审批”工作机制，逐步减少涉

企行政审批事项，实现 3 个工作日完成企业开办。建设政务

服务“一网通办”、数字政务一体化平台，推动“互联网+政务

服务”向基层延伸，实现 90%以上依申请行政权力事项和公

共服务事项“最多跑一次”。

激发和保护企业家精神。积极构建“亲”“清”新型政

商关系，健全领导干部联系服务企业工作制度，积极作为、

靠前服务、排忧解难。始终把企业家作为创新和发展的中坚

力量，激发各类市场主体活力，营造稳定公平透明、可预期

的营商环境，让广大企业家放心投资、舒心创业、安心发展。

（九）坚定不移深化改革扩大开放，增强发展动力活力

扎实推进重点领域改革。做好国资国企改革，推进国有

企业高质量发展。深化投融资体制改革，支持市属国有企业

发行债券融资。推进商事制度改革，全面实施市场准入负面

清单制度。开展农村集体产权制度改革，推进柳城县、鹿寨

县和北部生态新区县级农村产权流转交易中心试点，全面完

成农村集体资产清产核资。深化教育文化、医疗卫生、社保

养老等领域改革，使改革措施精准对接发展所需、基层所盼、

民生所向。

- 20 -

完善对外交通体系。加快推进已纳入广西铁路建设“十

三五”规划的柳州—广州铁路、河池—柳州—贺州城际高铁、

柳州—三江城际铁路报批工作，争取早日获批开工建设。全

面完成柳州站站房扩建工程和柳州动车存车场建设，加快湘

桂铁路柳州地区改建、涪陵至柳州、黔桂铁路扩能等铁路前

期工作。实施桂林至柳城、融水至河池、柳州经合山至南宁

高速公路以及城市周边 5 个高速公路进出口建设，推动阳朔

至鹿寨高速公路建成通车。推进红花二线船闸建设，开展柳

州新机场、新高铁站选址研究，打造立体式、智能化的综合

交通枢纽。

加快构建开放型经济体系。建立企业“走出去”联盟，支

持上汽通用五菱、柳工等企业参与“一带一路”国家地区共建

生产制造基地。继续新增国际航班和联接粤港澳大湾区航

线，推进柳州航空口岸正式开放，力争柳州保税物流中心实

现封关运营。主动参与珠江—西江经济带、粤港澳大湾区、

西部陆海新通道分工协作，加快建设粤桂黔高铁经济带合作

试验区（柳州）广西园。

（十）坚持以人民为中心，全力提高保障和改善民生水平

健全社会保障体系。做好高校毕业生、就业困难人员、

农村转移劳动力三大群体就业工作，实现城镇新增就业 6 万

人以上、农村劳动力转移就业 6.5 万人以上。推进全民参保

计划，健全覆盖城乡居民的社会保障体系。建设养老服务业

综合改革试验区，健全城乡特困供养人员供养标准自然增长

机制，关爱保护农村留守儿童、城乡困境儿童，完善社会救

- 21 -

助、社会福利制度。实施新一轮棚户区改造，完成 1.1 万套

保障性住房改造及新建任务。

坚持教育优先发展。促进义务教育优质均衡发展，深化

集团化办学和县属轮教，加强学生应急安全、文明素养等教

育。建设一批公益幼儿园，争取学前三年毛入园率达 90%。

开展普及高中阶段教育攻坚，力争高中阶段毛入学率达 90%。

支持广西科技大学“双一流”建设、柳职院创建中国特色高水平

高职学校和启动本科层次职业学校试点建设、鹿山学院转设

柳州工学院。推进职业教育国际化，加快建设广西现代职业

教育改革发展示范区、广西职业技能公共实训基地。

推动文化事业和文化产业发展。精心组织庆祝建国 70

周年文化活动，创排具有柳州特色、柳州印记、柳州品质的

优秀文艺作品。启动东门历史文化街区保护整治三期工程，

加快三江侗族村寨申遗。实施重点文化项目工程，加快文化

产业示范园区建设。认真组织筹办国际水上狂欢节等各项活

动，深入开展“春花秋水·画卷柳州”宣传，讲好柳州故事、

传播柳州声音。

加快卫生健康事业发展。深化城市公立医院综合改革，

推进高水平医联体建设，完善分级诊疗体系。加快实施工人

医院新院、儿童医院三期建设，提升中医药发展水平。开展

全民健康素养促进行动，启动国家卫生城市复审工作，提高

疾病防控、妇幼健康服务能力，免费为特定从业人员进行体

检。强化食品药品安全监管，全力保障群众饮食用药安全。

推进全民健身与全民健康深度融合，让城市更加充满活力。

- 22 -

打造共建共治共享的社会治理体系。强化平安柳州建

设，把扫黑除恶专项斗争引向深入，保持严打高压态势。进

一步畅通信访渠道，完善矛盾纠纷调解机制。全面落实安全

生产责任制，坚决遏制重特大事故发生，提升防灾减灾、应

急救援能力。加强全民普法，开展“爱城爱家、从我做起”

文明活动，注重为民靠民惠民，积极创建全国文明城市，努

力争创全国民族团结进步示范市。

推动军民融合深度发展。发挥柳州制造业优势，主动对

接国防科工部门，积极支持、服务国防和军队现代化建设。

落实优抚安置政策，维护军人军属合法权益，扎实开展双拥

模范城创建。

三、不断提高政府施政能力和服务水平

做好今年政府工作，必须进一步加强政府自身建设，提

高推动发展的能力，强化贯彻落实执行力，努力建设人民满

意政府。

一是坚持把政治建设摆在首位。树牢“四个意识”，坚

定“四个自信”，坚决维护习近平总书记在党中央的核心和

在全党的核心地位，坚决维护党中央权威和集中统一领导，

把习近平新时代中国特色社会主义思想贯穿到政府工作的

全过程，深入开展“不忘初心、牢记使命”主题教育，以严

实作风推动政府工作。

二是坚持依法行政，严格履行政府职责。坚决落实中央、

- 23 -

自治区和市委各项部署要求，有序推进深化机构改革工作。

推行重大行政决策预公开制度，完善权力清单和责任清单，

加强审计监督，规范和改进督查检查考核机制，提升政府公

信力、执行力。

三是加强廉政建设，强化担当落实。持之以恒落实中

央“八项规定”精神，严肃查处“表态多调门高，行动少

落实差”行为，坚决杜绝“议而不决、决而不施、施而无效” 现象。强化预算绩效管理，规范公务用车、办公用房。持

续开展“市长接待日”“市长企业接待日”和领导公开大接访

工作，全力为民办好 10 件实事，进一步密切与人民群众的

血肉联系。

各位代表，在新的一年政府工作中，我们将坚决执行市

人大及其常委会决议决定，自觉接受市人大及其常委会的法

律监督、工作监督；积极支持政协履行政治协商、民主监督、

参政议政职能。认真办理人大代表议案、批评、意见、建议

和政协委员提案，提高办成率、满意率。广泛听取各民主党

派、工商联、无党派人士和人民团体意见建议，努力打造务

实、高效、廉洁、法治的政府。

各位代表，责任呼唤担当，使命引领未来。让我们紧密

团结在以习近平总书记为核心的党中央周围，按照建设壮美

广西、共圆复兴梦想的总目标总要求，坚定信心，攻坚克难，

奋力谱写新时代柳州高质量发展新篇章，以优异成绩庆祝新

中国成立 70 周年！
